# Combining the Performance Strengths of the Logistic Regression and Neural Network Models: A Medical Outcomes Approach

**DOI:** 10.1100/tsw.2003.35

**Published:** 2003-06-09

**Authors:** Wun Wong, Peter J. Fos, Frederick E. Petry

**Affiliations:** McKesson Health Solutions, 275 Grove Street, Suite 1-110, Newton, MA 02466, USA

**Keywords:** neural networks, logistic regression, ROC curves, congestive heart failure

## Abstract

The assessment of medical outcomes is important in the effort to contain costs, streamline patient management, and codify medical practices. As such, it is necessary to develop predictive models that will make accurate predictions of these outcomes. The neural network methodology has often been shown to perform as well, if not better, than the logistic regression methodology in terms of sample predictive performance. However, the logistic regression method is capable of providing an explanation regarding the relationship(s) between variables. This explanation is often crucial to understanding the clinical underpinnings of the disease process. Given the respective strengths of the methodologies in question, the combined use of a statistical (i.e., logistic regression) and machine learning (i.e., neural network) technology in the classification of medical outcomes is warranted under appropriate conditions. The study discusses these conditions and describes an approach for combining the strengths of the models.

### INTRODUCTION

The study of patient outcomes has become increasingly important in the effort to contain medical costs, streamline patient management, and codify practices. Such study has aided the recent efforts to implement disease management programs (i.e., the application of outcomes principles to the practices of healthcare providers). By being able to predict the likelihood of a patient event prior to its occurrence, a case manager may learn to forestall or delay the event.

To study the feasibility of such an approach, a patient population has been selected (Medicare beneficiaries suffering from congestive heart failure [CHF] with identifiable outcomes [mortality within a specified period after discharge]). We develop neural network models and a model based on the logistic regression method, compare these approaches, and recommend a possible combined approach.

Neural networks have been used extensively in many industries including health care. They have, for instance, been the subject of extensive study and application in biomedicine and have been used to create diagnostic aides, analyze medical images, and to develop drugs. However, there has been limited use in the management of health care and so we investigate the possibility of their use in this area here.

For instance, issues such as the quality and management of patient care, the allocation of scarce resources, and the funding and reimbursement of institutional and provider services have dominated our discussions of the health care delivery system for the last 20 years. Since the formulation, analysis, and hoped for conclusion of these issues is dependent on the accurate portrayal of patient and administrative data, it is imperative that such data be efficiently and accurately mined for their contribution to these issues. This study tests the presumption that the use of neural networks to predict patient outcomes is valid and that its accuracy is comparable to that of conventional statistical approaches.

### BACKGROUND

#### Neural Nets

A neural network (NN) is a software- and/or hardware-based system of interconnected nodes (or neurons) that "learns" by modifying the connection strengths (or weights) between its elements in order to match the input-output behavior of the network to the process or system being modeled[1]. NNs are frequently composed of many computational elements operating in parallel. These computational nodes or neurons are connected via weights that are typically adapted to improve the performance of the network.

NN models are developed via training; the process of weight adaptation as prescribed by a set of well-defined rules[2]. The most common forms of learning include: (1) supervised learning (e.g., backpropagation) and (2) unsupervised learning (e.g., Kohonen self-organizing NN). Supervised learning requires the pairing of an input vector with a target vector (a training pair). With supervised learning, an NN is trained so that the application of a set of vector inputs will produce the set of vector outputs. Often, the NN "learns" through the minimization of error between the actual and estimated outputs. This occurs over many training iterations (epochs) since: (1) the initial weights are unlikely to provide the desired outputs and (2) many training pairs may be presented to a network. Incremental adjustments are made to the weights of a network until they gradually converge on an optimal set of values. This paper compares the performance of two supervised learning techniques. The training algorithms include: (1) multilayer perceptron (MLP) or backpropagation and (2) radial basis function (RBF).

The backpropagation method[3] is a very popular training algorithm in which an input vector is disseminated through a network in a forward fashion and the corresponding target output (actual) compared with the network derived output (estimated). Typically, the difference between the actual and the estimated outputs are calculated and the weights adjusted so as to minimize the sum-squared error. The modified errors are propagated back to the preceding nodes and layers (excluding the input layer) whereby the weights attached are adjusted to further minimize the sum-squared error. This process is repeated for the entire training set or matrix. As such, the input vectors are applied sequentially, errors calculated, and weights adjusted for each vector until the error for the entire training set is either at an acceptably low level (determined by the investigator) or a predetermined training duration had been reached.

The RBF network[4] is a supervised, feed-forward NN with a single hidden layer. Unlike multilayer networks, which transform a weighted summation of inputs, radial basis networks determine the outputs (each of which represents a basis function) of the hidden layer by measuring the distance between the network input and the center (or centroid) of each RBF.

### Logistic Regression

Logistic regression can be characterized as a modeling approach, which can be used to describe the relationship of several independent variables to a dichotomous dependent variable[5]. As such it lends itself to the classification of a dichotomous outcome such as the presence and occurrence or absence and nonoccurrence of a disease or event.

The logistic function is a squashing function that transforms an input with a value between $\pm$ infinity, into an output in the range [0,1]. The function f(z) indicates the risk for the presence or absence of a disease or event

$$f(z) = \frac{1}{1 \pm e^{-z}}$$

where $z = b + w_1p_1 + w_2p_2 + \ldots w_kp_k$ is an index of combined risk factors. Since in the domain for z of + infinity, f(z) ranges from 0 to 1, it can clearly be used to describe the probability or risk of an event or disease occurring.

The S-shaped curve of the logistic function provides a mechanism to consider the impact of a threshold on the likelihood of an outcome or risk for disease. The shape of the curve indicates that the effect of z will be negligible in the limit to – infinity. However as z increases, its impact will increase until a threshold is reached and so the risk or likelihood of the event (disease) rises rapidly over an intermediate range of values and then remains extremely high as z tends to + infinity.

So the logistic function can be converted to a logistic model

$$P(x) = \frac{1}{1 + e^{-(\alpha + \sum_{1}^{k} \beta_i x_i)}}$$

$= P(D = 1|x_1, x_2, \ldots x_k)$, whereby D = 1 represents the occurrence of the disease.

### Congestive Heart Failure

CHF is described as a disease process associated with profound symptoms and a poor long-term prognosis[6,7]. Its symptoms are characterized by abnormalities of the left and right ventricular function and are generally accompanied by changes in neurohormonal regulation, effort intolerance, fluid retention, and decreased survival. CHF is neither rare nor benign. It is often terminal, occurring after all reserve capacity and compensatory mechanisms of the myocardium and peripheral circulation have been exhausted. For many patients, the predominant symptom of CHF is a reduction of functional capacity due to poor exercise tolerance resulting from limited cardiac reserve[8,9].

### DATA SOURCES

The abstract patient data used for model development are provided by Louisiana Health Care Review (LHCR). Note: all unique patient and provider identifiers have been deleted from the data set and replaced with integers randomly assigned to each individual. The data form a subset of the Medicare (Part A) patient discharge database (fiscal 1990 to 1994) and includes all Louisiana hospital beneficiaries who are residents of the state.

Because the data set contains longitudinal patient data, it can be used to track a patient's passage through the prospective payment system. The data set contains all admission records (CHF or otherwise) of Louisiana patients with at least one principal diagnostic code (the admitting diagnosis) of CHF (n = 53,289). In addition, a smaller clinical data set is used to supplement the analysis. The clinical data set contains information from the LHCR's statewide baseline review. There are 1,068 records within the clinical data set. A listing of the variables included within the data sets is provided in Appendix A.

The study population includes all Medicare (Part A) beneficiaries who were admitted to a Louisiana hospital during the fiscal years 1991 through 1994 (October 1, 1990 through September 30, 1994). The study sample includes all Louisiana residents (within the study population) with at least one recorded episode (a principal diagnostic code) of CHF.

There are 35,271 persons included in the study sample. Of these, 20,986 (59.5%) are female; 14,275 (40.5%) male. Within the study sample, 24,984 (70.8%) are classified as "white", 8,861 (25.1%) "black", and; 1,145 (3.3%) "other" or "unknown". The study sample ranges in age from 64 to 109 years of age.

The definition of CHF (as provided LHCR) will include the principal diagnostic codes shown in Table 1. It should be noted the definition differs from the New York Heart Association's (NYHA) classification of CHF[10].

**TABLE 1**
**CHF Codes**

| Diagnosis Codes | Description |
|---|---|
| 402.01 | Hypertensive Heart Disease, Malignant with CHF |
| 402.11 | Hypertensive Heart Disease, Benign with CHF |
| 402.91 | Hypertensive Heart Disease, Unspecified with CHF |
| 404.01 | Hypertensive Heart & Renal Disease, Malignant with CHF |
| 404.03 | Hypertensive Heart & Renal Disease, Malignant with CHF & Renal Failure |
| 404.13 | Hypertensive Heart & Renal Disease, Benign with CHF & Renal Failure |
| 404.91 | Hypertensive Heart & Renal Disease, Unspecified with CHF |
| 404.93 | Hypertensive Heart & Renal Disease, Unspecified with CHF & Renal Failure |
| 425.4 | Other Cardiomyopathies — Includes Congestive |
| 428.0 | Heart Failure, CHF |
| 428.1 | Heart Failure, Left Heart Failure — Includes Left Ventricular Failure |
| 428.9 | Heart Failure, Unspecified |
| 429.3 | Ill Defined Descriptions & Complications of Heart Disease, Cardiomegaly — Includes Dilation, Hypertrophy, Ventricular Dilation |
| 518.4 | Acute Edema of Lung, Unspecified, Acute Pulmonary Edema NOS; Pulmonary Edema, Postoperative |

### MODEL DEVELOPMENT

The study design is retrospective, correlational, and nonexperimental. We first surveyed the literature to identify a preliminary set of independent variables.

#### Training, Testing, and Validation of Logistic Regression Model

In this phase, outcome or dependent variables were created that identify the mortality of a beneficiary within 90 and 365 days of discharge. The outcome variables are dichotomous and identify survival or death within a specified period after discharge. The variables were coded 0 (event did not occur) and 1 (event did occur), respectively, i.e., patient did not die within a specified time period vs. patient did die within a specified time period after discharge. Where appropriate, both the claims and clinical data sets were linked to create a combined data set (n = 1,024).

In addition, dummy variables were developed for race (White, Black), principal diagnosis (CHF, Unspecified with CHF), principal procedure (No Procedure, Operations on the Cardiovascular System), left ventricular hypertrophy from EKG and past MI from EKG. Dummy variables were also created for EKG-related categories in order to account for instances where an EKG was not administered. Interaction terms for the sociodemographic variables age**,** sex, race, and Metropolitan Statistical Area (MSA) were also developed as possible candidates for the development of the logistic regression model(s).

Two subsets of the database were created for the training and testing of the logistic regression models. The training set was used explicitly for model development, while the testing set was used to compare the predictive performances of the various (90- and 365-day) logistic regression and NN models.

In order to develop models, variables of significance to be considered as candidates for model development have to be determined. So tests for association between the outcome and dependent variables were generated using chi-square (categorical variables) and the Student t test (continuous variables). A significance level of 0.2 was chosen for each test and results are shown in Tables 2 and 3, respectively.

The development of the "final" model was a three-step process involving the creation of two intermediary and a conclusive logistic regression model. A forward (conditional) stepwise approach was applied to the development of the models. The entry of variables was based on the significance of the score statistic (<0.05). The removal (or exit) of variables was based on the significance (<0.1) of the likelihood ratio statistic (using conditional parameter estimates). The method of contrast was the "Indicator" method. The method testifies to the absence or presence of membership within a category. The reference group is by default the last category.

The first intermediary model was generated by "entering" all first-order terms in Block 1 of the logistic regression model. All second-order terms were included in Block 2 of the model. A forward (conditional) stepwise approach was applied to Block 2 of the model (entry: 0.05; removal: 0.1).

The second intermediary model was generated by "entering" in Block 1 all first- and second-order terms relevant to Block 2 of the previous or first intermediary model. The remaining first-order terms were included in Block 2 of the model. A forward (conditional) stepwise approach was applied to Block 2 of the model (entry: 0.05; removal: 0.1).

The "final" or conclusive model was generated by entering: (1) all first- and second-order terms from Block 1 of the second intermediary model and (2) all second-order terms relevant to Block 2 of the second intermediary model (entry: 0.05; removal: 0.1).

**Table 2A**
**Mortality within 90/365 Days of Discharge — Pearson Chi-Square 90 Days**
**Bivariate Test of Independence (<0.2)**

| Variable | Value | DF | *p* |
|---|---|---|---|
| Cardiomyopathy on X-ray | 2.58606 | 1 | 0.10781 |
| Pleural effusion on X-ray | 10.94007 | 1 | 0.00094 |
| Discharge with ACE inhibitor | 29.65021 | 1 | 0.00000 |
| History of CABG | 2.32166 | 1 | 0.12758 |
| History of ischemic cardiomyopathy | 2.69313 | 1 | 0.10078 |
| History of hypertension | 2.41583 | 1 | 0.12011 |
| History of MI | 2.50583 | 1 | 0.11343 |
| History of renal disease | 2.47928 | 1 | 0.11535 |
| History of valvular heart disease | 6.76347 | 1 | 0.00930 |
| Indicator for rales on physical examination | 2.60122 | 1 | 0.10678 |
| Indicator for angina | 3.53947 | 1 | 0.05992 |
| Absence of left ventricular hypertrophy (EKG) | 2.44909 | 1 | 0.11759 |
| Presence of left ventricular hypertrophy (EKG) | 5.52327 | 1 | 0.01877 |
| Absence of MI (EKG) | 6.45249 | 1 | 0.01108 |
| Presence of MI (EKG) | 4.83719 | 1 | 0.02785 |
| Principal diagnosis (CHF) | 3.30803 | 1 | 0.06894 |
| Principal procedure (Ops on Cardvasc Syst) | 2.53491 | 1 | 0.11135 |

**Table 2B**
**Mortality within 90/365 Days of Discharge — Pearson Chi-Square 365 Days**
**Bivariate Test of Independence (<0.2)**

| Variable | Value | DF | *p* |
|---|---|---|---|
| Absence of left ventricular hypertrophy (EKG) | 3.46354 | 1 | 0.06274 |
| Presence of left ventricular hypertrophy (EKG) | 3.64885 | 1 | 0.05611 |
| Cardiomyopathy on X-ray | 5.09732 | 1 | 0.02396 |
| Pleural effusion on X-ray | 9.59183 | 1 | 0.00195 |
| Discharge with ACE inhibitor | 9.43953 | 1 | 0.00212 |
| History of ischemic cardiomyopathy | 3.47372 | 1 | 0.06235 |
| History of cardiomyopathy | 2.85043 | 1 | 0.09135 |
| History of hypertension | 4.97780 | 1 | 0.02567 |
| History of MI | 4.49899 | 1 | 0.03391 |
| History of renal disease | 2.86993 | 1 | 0.09025 |
| History of valvular heart disease | 7.68126 | 1 | 0.00558 |
| Symptoms of angina | 5.74095 | 1 | 0.01657 |
| Symptoms of fatigue | 4.25461 | 1 | 0.03914 |

Probabilities for the occurrence (or nonoccurrence) of the event were developed for the beneficiaries within the testing set and receiver operating characteristic (ROC) curves were used to identify the cut-off point. In addition, cross-tabulations were used to compare the predicted outputs of the logistic regression models and the actual occurrences of the event.

**Table 3A**
**Mortality within 90 Days of Discharge Student t Test for Independent Samples Shaded Area: Variables of Significance**

| Variable | *p* (Levene's Test for Variances) | Variance | DF | 2-Tail Significance | 95% CI for Diff |
|---|---|---|---|---|---|
| Age | 0.164 | Equal | 498 | 0.001 | (–4.896, –1.339) |
| Blood, urea, and nitrogen (BUN) | 0.000 | Equal | 490 | 0.000 | (–12.522, –6.456) |
| Creatinine | 0.006 | Equal | 492 | 0.023 | (–0.207, –0.016) |
| Potassium | 0.002 | Equal | 493 | 0.033 | (–0.295, –0.012) |
| Pulse | 0.402 | Unequal | 116 | 0.243 | (–8.122, 2.080) |
| Diastolic BP | 0.252 | Unequal | 131 | 0.000 | (7.358, 14.882) |
| Systolic BP | 0.431 | Unequal | 135 | 0.000 | (12.934, 26.087) |
| Left ventricular dimensions (diastole) | 0.813 | Unequal | 24 | 0.966 | (–0.601, 0.576) |
| Left ventricular dimensions (systole) | 0.725 | Unequal | 21 | 0.368 | (–0.972, 0.376) |
| Left ventricular assessment (ventriculogram) | 0.077 | Equal | 6 | 0.281 | (–17.031, 49.031) |
| Left ventricular assessment (MUGA) | N/A | N/A | N/A | N/A | N/A |
| Ejection fraction (assessment prior to admission) | 0.383 | Unequal | 19 | 0.788 | (–9.621, 12.498) |
| Ejection fraction (current echo) | 0.145 | Equal | 135 | 0.708 | (–8.127, 11.935) |
| Ejection fraction (current calculated) | 0.659 | Unequal | 21 | 0.146 | (–2.551, 16.099) |
| Fractional shortening | 0.937 | Unequal | 10 | 0.910 | (–8.336, 9.248) |
| Length of stay | 0.000 | Equal | 498 | 0.007 | (–2.294, –0.370) |
| Total charge | 0.001 | Equal | 498 | 0.001 | (–4818, –1151) |

### Development of the Neural Network

Predictors identified by the previous models were used to create the NNs. They provide the input layer of the NN — the output node will comprise the patient outcome (it is binary). The following training algorithms were applied to the training set: (1) MLP or backpropagation and (2) RBF.

The choice of activation functions, number of hidden layers and nodes, and the final mean-squared error at termination were recorded. Random initialization of the weights was performed during model development. As with the development of the logistic regression models, the probabilities of the occurrence (or nonoccurrence) of the event were developed for the beneficiaries within the testing set and ROC curves were used to identify the probability cut-off point. Again cross-tabulations compared predicted outputs of the neural network models and actual event occurrences.

**Table 3B**
**Mortality within 360 Days of Discharge Student t Test for Independent Samples Shaded Area: Variables of Significance**

| Variable | *p* (Levene's Test for Variances) | Variance | DF | 2-Tail Significance | 95% CI for Diff |
|---|---|---|---|---|---|
| Age | 0.051 | Equal | 498 | 0.017 | (–3.276, –0.326) |
| Blood, urea, and nitrogen (BUN) | 0.000 | Equal | 490 | 0.000 | (–10.732, –6.548) |
| Creatinine | 0.001 | Equal | 490 | 0.001 | (–0.202, –0.051) |
| Potassium | 0.003 | Equal | 493 | 0.001 | (–0.305, –0.074) |
| Pulse | 0.038 | Equal | 498 | 0.169 | (–6.786, 1.191) |
| Diastolic BP | 0.628 | Unequal | 326 | 0.000 | (4.869, 11.231) |
| Systolic BP | 0.291 | Unequal | 363 | 0.000 | (10.746, 21.569) |
| Left ventricular dimensions (diastole) | 0.884 | Unequal | 70 | 0.999 | (–0.394, 0.394) |
| Left ventricular dimensions (systole) | 0.233 | Unequal | 58 | 0.427 | (–0.735, 0.316) |
| Left ventricular assessment (ventriculogram) | N/A | N/A | N/A | N/A | N/A |
| Left ventricular assessment (MUGA) | 0.727 | Unequal | 11 | 0.925 | (–15.550, 16.978) |
| Ejection fraction (assessment prior to admission) | 0.360 | Unequal | 48 | 0.326 | (–4.131, 12.195) |
| Ejection fraction (current echo) | 0.608 | Unequal | 56 | 0.979 | (–7.612, 7.819) |
| Ejection fraction (current calculated) | 0.306 | Unequal | 60 | 0.353 | (–3.638, 10.047) |
| Fractional shortening | 0.039 | Equal | 73 | 0.529 | (–7.043, 3.652) |
| Length of stay | 0.273 | Unequal | 296 | 0.131 | (–1.473, 0.191) |

### COMPARING PERFORMANCES OF LOGISTIC REGRESSION AND NEURAL NETWORK MODELS

The models were used to predict outcomes within the testing set. All ROC curves were reviewed and analyzed in order to compare the predictive performances of the various logistic regression and NN models. The measures of comparison include the following: overall accuracy, sensitivity, specificity, and Area Under Curve (Az).

#### Logistic Regression Model

We used several tests to evaluate the 90- and 365-day logisitic regression models. They include likelihood, Hosmer–Lemeshow test, ROC curves, and a z statistic. We first present a detailed discussion and results for the 90-day model and then a synopsis of the 365-day results.

### 90-Day Model

A sample of 484 cases was used to develop the 90-day logistic regression model. The variables (including interaction terms) that were identified as significant ($p < 0.05$) to the prediction of death within 90 days of discharge are seen in the Table 4A. The variable age was also included in order to develop the interaction terms: age by absence of left ventricular hypertrophy (EKG); age by presence of left ventricular hypertrophy (EKG).

**Table 4A**
**Significant Variables: Logisitic Regression Model — 90 Days**

| Variables | Significance *p* |
|---|---|
| Absence of left ventricular hypertrophy | *p* = <0.01 |
| Presence of left ventricular hypertrophy | *p* = <0.05 |
| Pleural effusion on X-ray | *p* = <0.01 |
| Blood, urea, and nitrogen (BUN) | *p* = <0.001 |
| Creatinine | *p* = <0.05 |
| Discharge with ACE inhibitor | *p* = <0.001 |
| Presence of MI (EKG) | *p* = <0.01 |
| History of valvular heart disease | *p* = <0.05 |
| Systolic BP | *p* = <0.01 |
| Age by absence of left ventricular hypertrophy (EKG) | *p* = <0.01 |
| Age by presence of left ventricular hypertrophy | *p* = <0.025 |

The certainty of the observed results (given the parameters of the model) is identified as the likelihood. Since likelihood <1, it is customary to use –2 multiplied by the log of the likelihood, or –2 log likelihood (–2LL) as a measure of model fit. The likelihood of a perfect fit for a model (to its data set) is 1 (the value of –2LL is 0). The –2LL for the 90-day logistic regression model (constant only) is 452.50. The –2LL for the model is 338.83; as the model improves, the value of –2LL decreases.

The model chi-square measures the difference between the –2LL for the model with constant only and the –2LL for the current model. It tests the null hypothesis that the coefficients for variables added at the last step are 0. Note: measure is comparable to the overall F statistic in linear regression. The model chi-square for the 90-day logistic regression model is 113.67 with 12 degrees of freedom. Since the test statistic is significant ($p < 0.0000$), we can reject the null hypothesis.

Goodness of fit measures indicate how well a model fits data. So next we used the Hosmer-Lemeshow test, which is a variant of the goodness of fit statistic[11]. It tests the null hypothesis that the model provides a poor fit to the data; it should be noted that if a model fits well with its data, the difference between the observed and predicted values based on the fitted model will be small and (as a consequence) the goodness of fit statistic will be nonsignificant. The Hosmer-Lemeshow statistic for the 90-day model is 8.928 with $p > 0.3$ where a small $p$ value ($p < 0.1$) would have indicated a poor fit between the observed and expected outcomes as seen in Table 5A. Table 6A shows the distribution of probabilities for the test.

An ROC curve[12] was generated to assess the predictive performance of the 90-day logistic regression model against the testing set of 294 cases (see Fig. 1A). Cut-offs, which maximized the overall accuracy of the model, were identified and using a cut-off of 0.10, the accuracy of the

### Table 5A
## Mortality within 90/365 Days of Discharge Logistic Regression Model. Calculation of Hosmer-Lemeshow Statistic — 90 Days

**Goodness of fit (Hosmer-Lemeshow test)**

**Observed counts**

| Died 90 | 1 | 2 | 3 | 4 | 5 | 6 | 7 | 8 | 9 | 10 | Total |
|---|---|---|---|---|---|---|---|---|---|---|---|
| 1 | 0 | 1 | 3 | 1 | 5 | 8 | 11 | 10 | 12 | 34 | 85 |
| 0 | 49 | 48 | 46 | 48 | 44 | 41 | 38 | 39 | 37 | 16 | 406 |
| Total | 49 | 49 | 49 | 49 | 49 | 49 | 49 | 49 | 49 | 50 | 491 |

**Expected Counts**

| Died 90 | 1 | 2 | 3 | 4 | 5 | 6 | 7 | 8 | 9 | 10 | Total |
|---|---|---|---|---|---|---|---|---|---|---|---|
| 1 | 0.53292 | 1.1393 | 1.76513 | 2.68127 | 3.9885 | 5.56195 | 8.26746 | 11.99561 | 17.61442 | 31.45355 | 85.00011 |
| 0 | 48.46708 | 47.8607 | 47.23487 | 46.31873 | 45.0115 | 43.43805 | 40.73254 | 37.00439 | 31.38558 | 18.54645 | 405.99989 |
| Total | 49 | 49 | 49 | 49 | 49 | 49 | 49 | 49 | 49 | 50 | 491 |

**(O - E)^2 / E**

| Died 90 | 1 | 2 | 3 | 4 | 5 | 6 | 7 | 8 | 9 | 10 | Total |
|---|---|---|---|---|---|---|---|---|---|---|---|
| 1 | 0.53292 | 0.0170319 | 0.8639046 | 1.0542276 | 0.2565206 | 1.0687057 | 0.9031522 | 0.3319931 | 1.7895402 | 0.2061582 | 7.0241541 |
| 0 | 0.0058597 | 0.0004054 | 0.0322834 | 0.0610265 | 0.0227305 | 0.1368406 | 0.1833123 | 0.1076213 | 1.0043374 | 0.3496307 | 1.9040477 |
| Total | 0.5387797 | 0.0174374 | 0.896188 | 1.1152541 | 0.279251 | 1.2055463 | 1.0864645 | 0.4396143 | 2.7938776 | 0.5557889 | 8.9282018 |

Test statistic follows a chi-square distribution with 8 df
p-value = 0.348389 indicating good fit
A small p-value (< 0.10) would indicate expected counts far from observed -> poor fit.

to get p-value : use CHIDIST(calc sum of (O-E)^2/E , degrees of freedom) = CHIDIST(9.49372,8)

### Table 6A
## Probability Distribution of Outcomes for 90-Day Logistic Regression Model — Administrative and Clinical Data Testing Set

| Probability of Death | Death within 90 Days of Discharge (Actual) | Survived within 90 Days of Discharge (Actual) | Total |
|---|---|---|---|
| 0.00–0.09 | 11 | 240 | 251 |
| 0.10–0.19 | 18 | 75 | 93 |
| 0.20–0.29 | 11 | 42 | 53 |
| 0.30–0.39 | 5 | 25 | 30 |
| 0.40–0.49 | 8 | 12 | 20 |
| 0.50–0.59 | 13 | 7 | 20 |
| 0.60–0.69 | 5 | 4 | 9 |
| 0.70–0.79 | 8 | 1 | 9 |
| 0.80–0.89 | 4 | 0 | 4 |
| 0.90–1.00 | 2 | 0 | 2 |
| Total | 85 | 406 | 491 |

model was 43% (sensitivity: 77%; specificity: 36%). Note: cut-offs may be manipulated to determine a sufficiently high sensitivity while maintaining an acceptably low false positive rate (1 - specificity). The area under the ROC curve (Az) was determined. The calculation of the Az and its standard error is based on the independence or correlation of the sample(s) under study. The comparison of independent samples utilizes the Mann-Whitney U statistic to calculate the Az and its standard error. The comparison of correlated samples (test values derived from same sample,

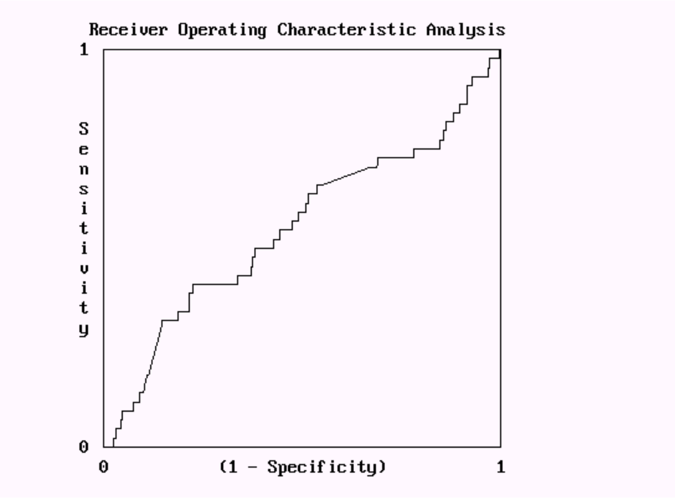

**FIGURE 1A.** ROC curve mortality within 90 days of discharge logistic regression. Test data: ______ Az: 0.55.

**Table 7A**
**Model Performance Comparison of Area Under the Curve: Administrative and Clinical Data Testing Set — 90-Day Models**

| Model | AZ | 95% CI | SE | One-Tail *p* | Two-Tail *p* |
|---|---|---|---|---|---|
| Log | 0.55 | (0.47, 0.65) | 0.0456 | 0.1237 | 0.2474 |
| MLP | 0.55 | (0.45, 0.65) | 0.0517 | 0.1397 | 0.2795 |
| BB | 0.66 | (0.57, 0.75) | 0.0446 | 0.0003 | 0.0007 |
| RBF | 0.65 | (0.56, 0.75) | 0.0479 | 0.0006 | 0.0012 |

equal sample size, cases in same order) utilizes the degree of correlation of the curves in order to increase the statistical power of the comparison[13]. Both approaches were used to compare the performances of the various models under consideration.

The Mann-Whitney U statistic has a well-characterized distribution that approaches normality as sample size increases. This feature enables the use of the z statistic to determine the degree to which the test or predictive model is superior to the "line of no information" (Az = 0.5). It tests the null hypothesis that the observed Az was obtained by chance. One- and two-tailed *p* values are then generated by way of the z statistic.

A z statistic was calculated by dividing the difference between the Az and the line of no information with the standard error of the Mann-Whitney U statistic in order to determine the degree to which the model is superior to the line of no information. It tests the null hypothesis that the Az was obtained by chance. One- and two-tailed *p* values were then generated by way of the z statistic. The Az for the 90-day logistic regression model was 0.55 (one-tail *p*: 0.1237; two-tail *p*: 0.2474). Since the test statistic is nonsignificant, we cannot reject the above null hypothesis (see Table 7A for detail).

### 365-Day Model

Our analysis follows that for the 90-day model above. A sample of 485 cases was used to develop the 365-day logistic regression model and the variables identified as significant ($p < 0.05$) to the prediction of death within 365 days of discharge are in Table 4B. Again the variable age was also included in order to develop the interaction terms.

**Table 4B**
**Significant Variables: Logisitic Regression Model — 365 Days**

| Variables | Significance *p* |
|---|---|
| Absence of left ventricular hypertrophy (EKG) | *p* = <0.05 |
| Cardiomyopathy on X-ray | *p* = <0.025 |
| Pleural effusion on X-ray | *p* = <0.025 |
| Blood, urea, and nitrogen (BUN) | *p* = <0.001 |
| Symptoms of angina | *p* = <0.025 |
| History of MI | *p* = <0.025 |
| Pulse | *p* = <0.001 |
| History of valvular heart disease | *p* = <0.01 |
| Diastolic BP | *p* = <0.001 |
| Age by absence of left ventricular hypertrophy (EKG) | *p* = <0.05 |
| Age by cardiomyopathy on x-ray | *p* = <0.05 |
| Age by symptoms of angina | *p* = <0.025 |

Here the –2LL for the 365-day logistic regression model (constant only) is 609.26 and the –2LL for the model is 494.77. The model chi-square for 365 days is 114.50 with 14 degrees of freedom. Since this test statistic is significant ($p < 0.0000$), we can reject the null hypothesis as for 90 days.

The Hosmer-Lemeshow statistic now is 7.06 with $p > 0.5$ (a small $p$ value [$p < 0.1$]) again indicating a poor fit between the observed and expected outcomes in Table 5B. Goodness of fit results are in Table 6B.

The ROC curve assessed the 365-day logistic regression model against the testing set of 256 cases (see Fig. 1B). With a cut-off of 0.41, the overall accuracy of the model was 57% (sensitivity: 70%; specificity: 50%).

One- and two-tailed $p$ values were generated again and the Az for the 365-day logistic regression model was 0.60 (one-tail $p$: 0.0048; two-tail $p$: 0.0096). Since the test statistic is significant, we can reject the null hypothesis. Table 7B illustrates this in contrast to the 90-day case.

### Neural Network Models

To develop the models, the variables significant ($p < 0.05$) to the prediction of mortality within 90/365 days of discharge were used as input to develop the respective MLP and RBF models (Table 8A). A training set of 152 cases was used to train the 90-day NN models and 285 cases for the 365-day models. A validation set of 18 cases was used to cross-validate the performance of the models during training for the 90-day model and 32 for the 365-day model.

Two layers (including the hidden and output layers) were used to develop the MLP or backpropagation network. The number of nodes within each layer was selected automatically. The number of nodes within the input layer total 18 and 4 in the hidden layer for 90 days and 21 and 5, respectively, for 365 days. The activation function for the hidden layer is a tangent sigmoid and the activation function for the output layer is linear.

**Table 5B**
**Mortality within 90/365 Days of Discharge Logistic Regression Model. Calculation of Hosmer-Lemeshow Statistic — 365 Days**

**Goodness of fit (Hosmer-Lemeshow test)**

**Observed counts**

| Died 365 | 1 | 2 | 3 | 4 | 5 | 6 | 7 | 8 | 9 | 10 | Total |
|---|---|---|---|---|---|---|---|---|---|---|---|
| 1 | 2 | 6 | 5 | 8 | 16 | 13 | 20 | 21 | 22 | 41 | 154 |
| 0 | 46 | 42 | 43 | 40 | 32 | 35 | 28 | 27 | 26 | 10 | 329 |
| Total | 48 | 48 | 48 | 48 | 48 | 48 | 48 | 48 | 48 | 51 | 483 |

**Expected Counts**

| Died 365 | 1 | 2 | 3 | 4 | 5 | 6 | 7 | 8 | 9 | 10 | Total |
|---|---|---|---|---|---|---|---|---|---|---|---|
| 1 | 2.57008 | 4.86842 | 6.89034 | 9.04235 | 11.21979 | 14.21217 | 17.35373 | 21.57054 | 27.0023 | 39.36049 | 154.09021 |
| 0 | 45.42992 | 43.13158 | 41.10966 | 38.95765 | 36.78021 | 33.78783 | 30.64627 | 26.42946 | 20.9977 | 11.63951 | 328.90979 |
| Total | 48 | 48 | 48 | 48 | 48 | 48 | 48 | 48 | 48 | 51 | 483 |

**(O - E)^2 / E**

| Died 365 | 1 | 2 | 3 | 4 | 5 | 6 | 7 | 8 | 9 | 10 | Total |
|---|---|---|---|---|---|---|---|---|---|---|---|
| 1 | 0.1264518 | 0.2630162 | 0.518608 | 0.1201561 | 2.0366163 | 0.1033872 | 0.4035297 | 0.0150908 | 0.926699 | 0.0682917 | 4.5818467 |
| 0 | 0.0071537 | 0.0296876 | 0.0869233 | 0.0278891 | 0.6212691 | 0.0434877 | 0.2285024 | 0.0123164 | 1.1917022 | 0.230937 | 2.4798684 |
| Total | 0.1336055 | 0.2927038 | 0.6055312 | 0.1480452 | 2.6578854 | 0.1468749 | 0.632032 | 0.0274072 | 2.1184012 | 0.2992286 | 7.0617151 |

Test statistic follows a chi-square distribution with 8 df
p-value = 0.529989 indicating good fit
A small p-value (< 0.10) would indicate expected counts far from observed -> poor fit.

to get p-value : use CHIDIST(calc sum of (O-E)^2/E , degrees of freedom) = CHIDIST(9.49372,8)

**Table 6B**
**Probability Distribution of Outcomes for 365-Day Logistic Regression Model — Administrative and Clinical Data Testing Set**

| Probability of Death | Death within 365 Days of Discharge (Actual) | Survived within 365 Days of Discharge (Actual) | Total |
|---|---|---|---|
| 0.00–0.09 | 4 | 68 | 72 |
| 0.10–0.19 | 17 | 96 | 113 |
| 0.20–0.29 | 21 | 57 | 78 |
| 0.30–0.39 | 26 | 45 | 71 |
| 0.40–0.49 | 24 | 29 | 53 |
| 0.50–0.59 | 15 | 21 | 36 |
| 0.60–0.69 | 17 | 7 | 24 |
| 0.70–0.79 | 12 | 4 | 16 |
| 0.80–0.89 | 15 | 2 | 17 |
| 0.90–1.00 | 5 | 0 | 5 |
| Total | 156 | 329 | 485 |

### *90-Day Model*

A total of 86 weights were generated by the 90-day MLP model. The conjugate gradient descent method was used to alter nodal weights during training; the initialization of weights was random. The conjugate gradient method measures the gradient of the error surface after each backward and forward propagation of the model. It then adjusts nodal weights in order to minimize the mean-square error. After training of the NN model is terminated, the root mean square (RMS) error was recorded. The RMS error for the 90-day MLP network was 0.52.

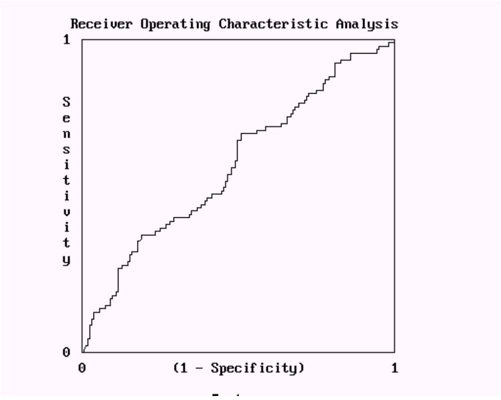

**FIGURE 1B**. ROC curve mortality within 365 days of discharge logistic regression. Test data: _____ AZ: 0.60.

**Table 7B**
**Model Performance Comparison of Area Under the Curve: Administrative and Clinical Data Testing Set — 365-Day Models**

| Model | AZ | 95% CI | SE | One-Tail *p* | Two-Tail *p* |
|---|---|---|---|---|---|
| Log | 0.60 | (0.53, 0.67) | 0.0370 | 0.0048 | 0.0096 |
| MLP | 0.69 | (0.62, 0.76) | 0.0348 | 0.0000 | 0.0000 |
| BB | 0.65 | (0.58, 0.72) | 0.0357 | 0.0000 | 0.0001 |
| RBF | 0.67 | (0.61, 0.75) | 0.0353 | 0.0000 | 0.0000 |

**Table 8A**
**Significant Variables: Neural Network Models — 90 Days**

| Variables | Significance *p* |
|---|---|
| Absence of left ventricular hypertrophy | $p$ = <0.01 |
| Presence of left ventricular hypertrophy | $p$ = <0.05 |
| Pleural effusion on X-ray | $p$ = <0.01 |
| Blood, urea, and nitrogen (BUN) | $p$ = <0.001 |
| Creatinine | $p$ = <0.05 |
| Discharge with ACE inhibitor | $p$ = <0.001 |
| Presence of MI (EKG) | $p$ = <0.01 |
| History of valvular heart disease | $p$ = <0.05 |
| Systolic BP | $p$ = <0.01 |
| Age by absence of left ventricular hypertrophy (EKG) | $p$ = <0.01 |
| Age by presence of left ventricular hypertrophy | $p$ = <0.025 |

Two layers (including the hidden and output layers) were used to develop the 90-day RBF network. The number of nodes within the input layer total 18. The activation function for the output layer is a spline function whereby f(x) = $\mathbf{d}^2\log\mathbf{d}$ (where **d** = distance of x from a centroid). The positional strategy for the initial centroids was based on a sampling of data points and the distance measure in use was Euclidean. A total of 30 centroids (reflecting the optimal number of centers within the data set) were generated by the 90 RBF model. Note: the number of centroids chosen can range from 5 to 50. The RMS error for the 90-day RBF network was 0.62.

A ROC curve was again used to assess the performance of the 90-day MLP model (see Fig. 2A). Using a cut-off of 0.82, the accuracy of the model was 72% (sensitivity: 41%; specificity: 78%). The Az for the 90-day MLP network was 0.55 (one-tail *p*: 0.1397; two-tail *p*: 0.2795). Since the test statistic is nonsignificant, we cannot reject the null hypothesis (see Table 7A for details). For the 90-day RBF model (see Fig. 2B) with a cut-off of 0.51, the accuracy now was 62% (sensitivity: 68%; specificity: 61%). The Az was 0.65 (one-tail *p*: 0.0006; two-tail *p*: 0.0012), and now since the test statistic is significant, the null hypothesis is rejected (Table 7A).

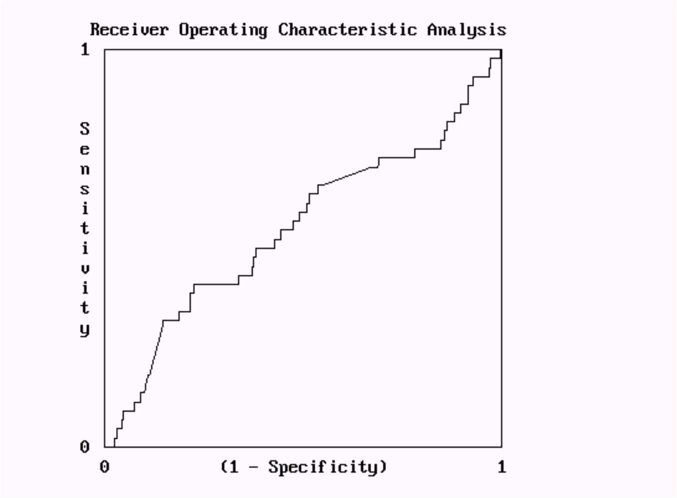

**FIGURE 2A**. ROC curve mortality within 90 days of discharge MLP. Test data: _____ Az: 0.55.

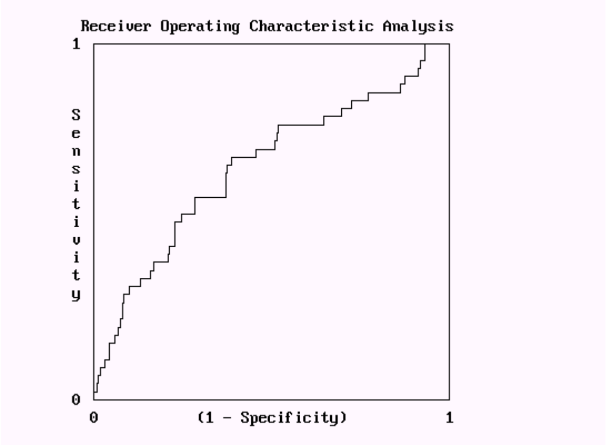

**FIGURE 2B**. ROC curve mortality within 90 days of discharge RBF. Test data: _____ AZ: 0.65.

### *365-Day Model*

The significant variables for the 365-day model are seen in Table 8B and again, age developed the interaction terms.

**Table 8B**
**Significant Variables: Neural Network Models — 365 Days**

| Variables | Significance *p* |
|---|---|
| Absence of left ventricular hypertrophy (EKG) | $p$ = <0.05 |
| Cardiomyopathy on X-ray | $p$ = <0.025 |
| Pleural effusion on X-ray | $p$ = <0.025 |
| Blood, urea, and nitrogen (BUN) | $p$ = <0.001 |
| Symptoms of angina | $p$ = <0.025 |
| Symptoms of fatigue | $p$ = <0.025 |
| History of MI | $p$ = <0.025 |
| Pulse | $p$ = <0.001 |
| History of valvular heart disease | $p$ = <0.01 |
| Diastolic BP | $p$ = <0.001 |
| Age by absence of left ventricular hypertrophy (EKG) | $p$ = <0.05 |
| Age by cardiomyopathy on X-ray | $p$ = <0.05 |
| Age by symptoms of angina | $p$ = <0.025 |

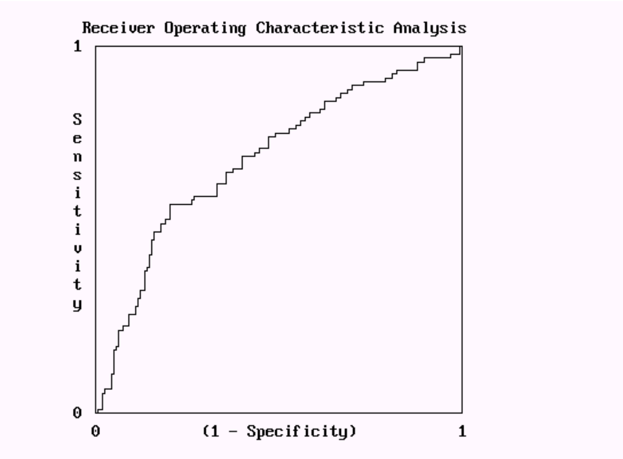

**FIGURE 3A.** ROC curve mortality within 90 days of discharge MLP. Test data: _____ AZ: 0.69.

A total of 122 weights were used for the MLP model, again with conjugate gradient descent for training with random initialization of weights. The RMS error after training was 0.61.

As for the 90-day example, two layers were used to develop the 365-day RBF network with a similar approach. A total of 20 centroids were generated and the RMS error here was 0.59.

The ROC curve for the 365-day MLP model is seen in Figure 3A. Here with a cut-off of 0.58, the accuracy of the model was 72% (sensitivity: 57%; specificity: 80%). The Az for was 0.69 (one-tail $p$: 0.0000; two-tail $p$: 0.0000) and the null was rejected due the significance of the test statistic (Table 7B).

For the RBF, the ROC curve is in Figure 3B. Now with a cut-off of 0.53, the model's accuracy was 67% (sensitivity: 58%; specificity: 72%) and the Az was 0.67 (one-tail $p$: 0.0000; two-tail $p$: 0.0000). Again the test statistic is significant, and we can reject the null hypothesis as seen in Table 7B.

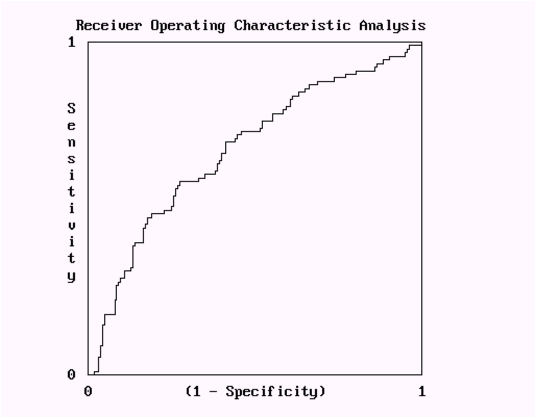

**FIGURE 3B**. ROC curve mortality within 90 days of discharge multilayer RBF. Test data: _____ AZ: 0.67.

## RESULTS

### Comparison of Logistic Regression and Neural Network Models

A comparison of the 90-day logistic regression and NN models reveals the following: (1) we accept (do not reject) the null hypothesis that no difference exists between the Az of the logistic regression and MLP network (chi-square = 0.0038; df = 1; one-tail $p$ = 0.4753; two-tail $p$ = 0.9506); (2) we reject the null hypothesis that no difference exists between the Az of the logistic regression and RBF network (chi-square = 2.2177; df = 1; one-tail $p$ = 0.0682; two-tail $p$ = 0.1364).

Similarly for the 365-day cases we see: (1) we reject the null hypothesis that no difference exists between the Az of the logistic regression and MLP network (chi-square = 5.8766; df = 1; one-tail $p$ = 0.0077; two-tail $p$ = 0.0153); (2) we reject the null hypothesis that no difference exists between the Az of the logistic regression and RBF network (chi-square = 4.2225; df = 1; one-tail $p$ = 0.0200; two-tail $p$ = 0.0399).

Based on the findings for the study population, we accept (do not reject) the assertion stating that NNs represent a valid approach to predicting patient outcomes. Given the parameters of the study, it has also been shown that NNs can outperform the logistic regression model in terms of sample prediction.

Table 9 provides a comparison of the sensitivity, specificity, positive predictive value, and negative predictive value of the various models.

### Limitations

The study has determined that: (1) NNs represent a valid approach to predicting the mortality of the study population within a specified period after discharge and (2) NNs are able to outperform the logistic regression in terms of sample prediction.

Several limiting factors should be taken into account when considering the results of this study. These limitations relate to the study's methodological approach, the paucity of clinical predictors, the reliance on default parameters in the development of the NN models, and the nature of the disease process identified as CHF.

For example, the study's approach to the development of the logistic regression model carries inherent limitations that may hinder its performance. It should be noted that a logistic regression model forces a linear relationship between a continuous predictor and the predicted log odds of its

**Table 9A**
**Model Performance Comparison of Sensitivity and Specificity Administrative and Clinical Data Testing Set — 90-Day Models**

| Model | Cut-Off | Overall | Sens | 95% CI | Spec | 95% CI | PPV | NPV |
|---|---|---|---|---|---|---|---|---|
| Log | 0.10 | 0.43 | 0.77 | (0.6216, 0.8853) | 0.36 | (0.3043, 0.4270) | 0.18 | 0.90 |
| MLP | 0.82 | 0.72 | 0.41 | (0.2634, 0.5675) | 0.78 | (0.7192, 0.8261) | 0.24 | 0.88 |
| BB | 0.38 | 0.54 | 0.77 | (0.6216, 0.8853) | 0.50 | (0.4363, 0.5637) | 0.21 | 0.93 |
| RBF | 0.51 | 0.62 | 0.68 | (0.5242, 0.8139) | 0.61 | (0.5486, 0.6727) | 0.24 | 0.92 |

n = 294.

**Table 9B**
**Model Performance Comparison of Sensitivity and Specificity Administrative and Clinical Data Testing Set — 365-Day Models**

| Model | Cut-Off | Overall | Sens | 95% CI | Spec | 95% CI | PPV | NPV |
|---|---|---|---|---|---|---|---|---|
| Log | 0.41 | 0.57 | 0.70 | (0.5950, 0.7897) | 0.50 | (0.4118, 0.5702) | 0.44 | 0.74 |
| MLP | 0.58 | 0.72 | 0.57 | (0.4631, 0.6722) | 0.80 | (0.7276, 0.8564) | 0.62 | 0.77 |
| BB | 0.95 | 0.65 | 0.04 | (0.0118, 0.1065) | 0.99 | (0.9564, 0.9985) | 0.67 | 0.64 |
| RBF | 0.53 | 0.67 | 0.58 | (0.4738, 0.6822) | 0.72 | (0.6486, 0.7910) | 0.55 | 0.75 |

n = 256.

outcome variable. Information is lost when a nonlinear relationship is thus forced into a linear relationship. Since the logistic regression models contain continuous predictors that may harbor a nonlinear relationship with its outcome variable, the threat of such a loss must be acknowledged. If a nonlinear relationship does exist between a continuous predictor and its outcome variable, it is advisable to capture it by converting the continuous into categorical variables.

The paucity of clinical predictors is another limiting component of model development. Many of the variables available simply had too many missing values to be of use. Note: one example includes the numerous predictors that pertain to ejection fraction or its subjective assessment.

The predictors eventually selected via model development include historical (pertaining to the presence or absence of a condition), radiographic (pertaining to the presence or absence of a condition or a treatment regimen), and laboratory data (pertaining to BUN, creatinine, and potassium values); it should be noted that these values have the advantage of simplicity and availability. Little information was captured with respect to ejection fraction (a variable widely thought to be an indicator of poor health within a population suffering from CHF) or its colloraries.

The reliance on default parameters in the development of the NN models is another limiting factor of model development. In order to enforce a uniformity of approach to network development (and thus support the study's comparative efforts), it was decided to rely only on the default parameters available to the software program. As such, the optimization of each NN model was sacrificed for uniformity. It is anticipated that individual efforts to optimize the performance of each network will result in a marked improvement of their performances. This effort is recommended if the models are to be used within an applied setting.

The nature of CHF is another limiting component of model development. Rightly identified as a syndrome, its lack of specificity is reflected in the data and may have hindered model development.

### CONCLUSIONS

Neural networks represent a broad class of nonlinear regression (e.g., MLP or backpropagation network) and discriminant models (e.g., ADALINE); data reduction and nonlinear dynamical systems (e.g., Kohonen self-organizing network)[14]. They are oftentimes similar or identical to popular statistical techniques (e.g., generalized linear models, principal components, cluster analysis, polynomial regression), particularly if the emphasis were on the prediction, rather than the explanation of outcomes. Such similarities may account for their strengths. As such, they have much to offer by way of data analysis and classification.

It has been shown that given the parameters of the study and its sample, the NN methodology is superior to the logistic regression methodology in terms of sample prediction performance. It must be noted, however, that the use of the logistic regression methodology (or statistical methods in general) should not be overlooked. On the contrary the logistic regression methodology is capable of providing an explanation (either intuitive or explicit) regarding the relationship(s) between: (1) the outcome and the independent variables (multivariate analyses) and (2) the dependent variables (bivariate analyses). Also, statistical software programs can also be used to produce confidence intervals, prediction intervals, diagnostics, and various graphical displays — features that are rarely provided by NN technologies[14].

The study therefore recommends that the combined use of statistical and machine learning technologies in the classification of medical/patient outcomes is warranted when: (1) information regarding the relationship(s) between variables is/are unclear, i.e., the exercise of producing a logistic regression is helpful to the understanding of relationships (both statistical and clinical) between variables; and (2) improving the predictive performance of a model is critical for clinical or disease management purposes. It also recommends an approach that is similar to the study's methodology.

The study recommends that future research efforts should include the application of NN technology within a patient or disease management setting. In addition, other candidates for further research may include: (1) the prediction of continuous outcomes from recorded healthcare data, (2) the prediction of discrete outcomes from transient (or real time) healthcare data, and (3) the prediction of continuous outcomes from transient (or real time) healthcare data.

### BIOSKETCHS

**Dr. W. Wong** received a Doctorate of Public Health in 1998 from Tulane University Medical Center, School of Public Health and Tropical Medicine, New Orleans, LA. He currently holds the position of Director of Research and Development, CareEnhance Products at McKesson Health Solutions, Newton, MA. His work includes the application of data mining and knowledge discovery tools to large healthcare databases, and the development, testing, and validation of statistical and machine learning models. Using a variety of analytic tools (including those derived from disciplines such as statistics, economics, decision analysis, and computer science), he has been involved in a variety of projects including healthcare outcomes research, data mining, and predictive modeling.

**Dr. Peter J. Fos** currently holds the position of Chief Science Officer and Director of the Office of Science at the Mississippi State Department of Health, Jackson, MS. Dr. Fos was Full Professor of Clinical Sciences in the UNLV School of Dentistry, and served as Chair of the Department of Clinical Sciences from March 2002 until December 2002. He has held an Associate Professor position in Health Systems Management at Tulane University Medical Center, School of Public Health and Tropical Medicine from 1996–2002, and was Assistant Dean for Undergraduate Relations. Dr. Fos received a Master of Public Health in Health Systems Management in 1985 from Tulane University Medical Center, School of Public Health and Tropical Medicine, and a Ph.D. in Health Care Decision Analysis from Tulane University Graduate School in 1989. Since 1987, Dr. Fos has held a faculty position at Tulane University Medical Center, School of Public Health and Tropical Medicine.

His research interests include public health system planning and evaluation, managerial epidemiology, decision sciences, negotiation, clinical effectiveness, and outcomes research. He has published in the following journals: *Medical Decision Making*, *Decision Sciences*, *Journal of Health Administration Education*, S*ocio-economic Planning Sciences*, *Journal of Environmental Health*, *Hospital and Health Services Administration*, *Journal of Public Health Management and Practice*, *Health Care Management Science*, and *Salud Publica de Mexico*. He has written, with

David J. Fine, *Designing Health Care for Populations: Applied Epidemiology for Health Care Administration* (Jossey-Bass, April 2000).

Dr. Fos is a member of the American Dental Association, American Public Health Association, the Association of Health Services Research, Society of Medical Decision Making, and the Association of University Programs in Health Administration.

**Dr. Fred Petry** received B.S. and M.S. degrees in physics and a Ph.D. in computer and information science from the Ohio State University in 1975. He has been on the faculty of the University of Alabama in Huntsville and the Ohio State University and is currently a Full Professor in the Department of Electrical Engineering & Computer Science at Tulane University, New Orleans, LA. He is cofounder/director of the Center for Intelligent and Knowledge Based Systems (CIAKS) at Tulane and his recent research interests include representation of imprecision via fuzzy sets and rough sets in databases, GIS, and other information systems, and artificial intelligence including genetic algorithms. His research has been funded by NSF, NASA, DOE, NIH, various DOD agencies, and industry. He has directed 20 Ph.D. students in these areas in the past 10 years.

Dr. Petry has over 250 scientific publications including 100 journal articles/book chapters and 5 books written or edited. His monograph on fuzzy databases has been widely recognized as the definitive volume on this topic. He is currently an associate editor of *IEEE Transactions on Fuzzy Systems*, *Neural Processing Letters* and area editor of information systems for *Fuzzy Sets and Systems* and was general chairperson of FUZZ-IEEE '96. He was selected as an IEEE Fellow in 1996 and an IFSA fellow in 2003 for his research on the use of fuzzy sets for modeling imprecision in databases and was named the Tulane School of Engineering Outstanding Researcher for 2002.

## APPENDIX A

### Administrative Claims Data

The following variables are included within the claims data set (n = 157,432): patient identifier (arbitrary); facility identifier (arbitrary); Prospective Payment System (PPS) code; non-PPS claim code; record link; age; sex; race; Zip code; Louisiana resident; Metropolitan Statistical Area (MSA); urban/rural designation of hospital (HCFA); Diagnosis-Related Group (DRG); principal diagnosis group; secondary diagnosis group (1 to 9 diagnostic codes available); procedure code (1 to 6 diagnostic codes available); DRG price (price HCFA utilizes to assess payment); total charge (billing by hospital); reimbursement (amount paid to HCFA); Intensive Care Unit (ICU) stay; Coronary Care Unit (CCU) stay; in-patient death; admission date; (elapsed); discharge date (elapsed); date of death (elapsed); bad bill indicator; index; admission date (actual); discharge date (actual); date of death (actual).

### Clinical Data

The following variables are included within the clinical data set (n = 1,024): assessment of left ventricular systolic function prior to admission; method of past assessment of left ventricular function; ejection fraction from past assessment; past subjective assessment of left ventricular function (if ejection fraction missing); echocardiogram for current admission; ejection fraction from echocardiogram; subjective assessment of left ventricular function (if ejection fraction missing from echocardiogram); MUGA for current admission; left ventricular function from MUGA; subjective assessment of left ventricular function (if assessment missing from MUGA); left ventriculogram for current admission; left ventricular assessment from ventriculogram; subjective assessment of left ventricular function (if assessment missing from ventriculogram); left ventricular internal dimensions (diastole); left ventricular internal dimensions (systole); fractional shortening (calculated from left ventricular internal dimensions [diastole and systole]); ejection fraction (calculated from left ventricular internal dimensions [diastole and systole]); low ejection fraction indicator; admission blood pressure (systole); admission blood pressure (diastole); admission pulse; Blood, Urea, Nitrogen (BUN) admission value; admission creatinine value; admission potassium value; indicator for shortness of breath; indicator for dypsnea on exertion; indicator for othopnea; indicator for paroxysmal nocturnal dypsnea; indicator for fatigue; indicator for angina; indicator for S3 gallop from physical examination; indicator for displace point of maximum impulse on physical examination; indicator for rales on physical examination; history of CHF; history of ischemic heart disease; history of myocardial infarction; history of CABG; history of PTCA; history of renal disease; history of hypertension; history of diabetes; history of angina; history of atrial fibrillation; history of valvular heart disease; history of renovascular disease; history of adverse reaction to ACE inhibitors; history of cardiomyopathy; history of ischemic cardiomyopathy; history of nonischemic cardiomyopathy; history of hypertrophic cardiomyopathy; history of nonspecified cardiomyopathy; X-ray for current admission (yes/no); indicator for congestion on X-ray; indicator for edema on X-ray; indicator for pulmonary vascular congestion on X-ray; indicator for cardiomyopathy on X-ray; indicator for pleural effusion on X-ray; indicator that X-ray is unclear; second X-ray for current admission (yes/no); indicator for congestion on second X-ray; indicator for edema on second X-ray; indicator for pulmonary vascular congestion on second X-ray; indicator for cardiomyopathy on second X-ray; indicator for pleural effusion on second X-ray; EKG for current admission (yes/no); indicator for normal EKG; indicator for past MI from EKG; indicator for left ventricular hypertrophy from EKG; EKG rhythm; patient admitted with ACE inhibitor (yes/no); patient discharged with ACE inhibitor (yes/no).
